# Development of a Bioprocess for the Production of Cyclic Lipopeptides Pseudofactins With Efficient Purification From Collected Foam

**DOI:** 10.3389/fbioe.2020.565619

**Published:** 2020-11-23

**Authors:** Piotr Biniarz, Marius Henkel, Rudolf Hausmann, Marcin Łukaszewicz

**Affiliations:** ^1^Department of Biotechnology and Food Microbiology, Wrocław University of Environmental and Life Sciences, Wrocław, Poland; ^2^Department of Biotransformation, Faculty of Biotechnology, University of Wrocław, Wrocław, Poland; ^3^Department of Bioprocess Engineering (150 k), Institute of Food Science and Biotechnology, University of Hohenheim, Stuttgart, Germany

**Keywords:** biosurfactant production, cyclic lipopeptides (CLPs), *Pseudomonas fluorescens*, bioreactor, lipopeptide production, lipopeptide purification, foam fractionation

## Abstract

Microbial surfactants (biosurfactants) have gained interest as promising substitutes of synthetic surface-active compounds. However, their production and purification are still challenging, with significant room for efficiency and costs optimization. In this work, we introduce a method for the enhanced production and purification of cyclic lipopeptides pseudofactins (PFs) from *Pseudomonas fluorescens* BD5 cultures. The method is directly applicable in a technical scale with the possibility of further upscaling. Comparing to the original protocol for production of PFs (cultures in mineral salt medium in shaken flasks followed by solvent-solvent extraction of PFs), our process offers not only ∼24-fold increased productivity, but also easier and more efficient purification. The new process combines high yield of PFs (∼7.2 grams of PFs per 30 L of working volume), with recovery levels of 80–90% and purity of raw PFs up to 60–70%. These were achieved with an innovative, single-step thermal co-precipitation and extraction of PFs directly from collected foam, as a large amount of PF-enriched foam was produced during the bioprocess. Besides we present a protocol for the selective production of PF structural analogs and their separation with high-performance liquid chromatography. Our approach can be potentially utilized in the efficient production and purification of other lipopeptides of *Pseudomonas* and *Bacillus* origin.

## Introduction

Cyclic lipopeptides (CLPs) are a class of surface-active compounds of microbiological origin – biosurfactants (BS). According to the NORINE database, more than 950 CLPs, grouped in 145 families have been identified up to now (Flissi et al., 2016). A majority of them are produced by *Bacillus* and *Pseudomonas* strains, however, other microorganisms have been also reported as their possible sources (Caboche et al., 2008). Surfactins, produced by *B. subtilis*, are probably the best known and studied CLPs. Iturins, fengycins, and lychenisins are yet another CPLs families produced by *Bacillus* (Coutte et al., 2017). The list of potential natural functions of CLPs includes e.g., roles in antagonism, protection agents, *quorum sensing* molecules, chelators, and others (Raaijmakers et al., 2010), while the list of potential applications of *Bacillus* CLPs includes e.g., antimicrobial and anticancer drugs, cleansing agents, plant protection or bioremediation boosting agents (Banat et al., 2010; Mukherjee and Das, 2010; Li et al., 2019; Naughton et al., 2019). Similarly, *Pseudomonas*-derived CLPs exhibit even a larger diversity and are divided into several families: putisolvins, amphisins, viscosins, and others (Raaijmakers et al., 2010; Flissi et al., 2016; Götze and Stallforth, 2019). The *Pseudomonas*-derived CLPs are also highly active surfactants with a number of potential applications (Raaijmakers et al., 2010; Götze and Stallforth, 2019; Naughton et al., 2019).

Diversity of CLPs encompasses not only differences between families, but also heterogeneity within the family. CLPs of a given family are often produced as a mixture of structural analogs, and the differences between analogs include varying length and branching of hydrophobic moieties, as well as amino acid substitutions in the peptide core (Raaijmakers et al., 2006; de Bruijn et al., 2007; Biniarz et al., 2016). The diversity of CLPs is caused by the mechanism of their biosynthesis by NRPS (non-ribosomal peptide synthetases) complexes. The NRPS enzymes are organized into modules and each module is responsible for introducing one amino acid to the CLP molecule. Therefore, the size (length) and structure of a certain CLP depend on the modular organization of the NRPS complex. Mentioned diversity of CLPs is due not only to their synthesis by different NRPS complexes, but also to the substrate specificity of NRPS modules. The detailed information can be found in a number of excellent publications (Roongsawang et al., 2010). It is worth stressing, that even a minor modification in the CLP structure can lead to the significant changes in their activity and properties (Nguyen et al., 2010; Roongsawang et al., 2010). Due to the similarity of CLPs analogs, their purification is rather difficult. Therefore, most research on the properties of CLPs is carried on the mixtures of analogs, leaving the issue of structure-properties relationship of CLPs largely unaddressed (Raaijmakers et al., 2006; de Bruijn et al., 2007; Nguyen et al., 2010; Roongsawang et al., 2010).

The widespread use of BS is hindered by a troublesome production and purification, and relatively high costs of these processes (Coutte et al., 2017; Henkel et al., 2017). Over the years, different approaches have been tested to reduce the costs of BS manufacturing. The use of bioreactors seems to be especially promising, as it allows the processes to be performed in the industrial scale, while retaining precise control (Guez et al., 2008; Motta Dos Santos et al., 2016; Coutte et al., 2017). In the manufacturing process of BS, downstream processing is often mentioned as a critical step, generating approximately 60% of the total manufacturing costs (Chen et al., 2015; Coutte et al., 2017). There are two major methods used for BS recovery and purification: acid precipitation and liquid-liquid extraction. These methods are easy to apply in the laboratory scale, but their up-scaling can be problematic. Also, BS recovery and purity can be relatively low for these methods (Chen et al., 2007; Smyth et al., 2010; Biniarz et al., 2016; Coutte et al., 2017; Varjani and Upasani, 2017). Recently, ultrafiltration emerged as a promising BS-purification technique that can be applied in the industrial-scale production of BS. Ultrafiltration offers high recovery and purity levels of BS, together with the ease of up-scaling and the possibility of developing continuous processes. The limitations of ultrafiltration include for instance difficulties when working with high-viscosity samples or relatively high complexity of ultrafiltration setup (Chen et al., 2007; Coutte et al., 2010, 2013, 2017; Jauregi et al., 2013; Rangarajan and Clarke, 2016). Foam fractionation is another method proposed for the initial purification of BS. As surface active substances tend to accumulate at the phase interfaces, BS can be efficiently recovered with foam directly from the fermentation medium, by coupling a foam column to the bioreactor. Despite this, bioprocesses involving foam fractionation can be difficult to control as nutrients, autoinducer molecules, and/or bacterial cells can be depleted from a bioreactor vessel with overflowing foam (Coutte et al., 2017).

Pseudofactins (PFs) are a family of CLPs produced by the Arctic isolate *Pseudomonas fluorescens* BD5 (Janek et al., 2010). Four PF structural analogs were previously identified (PF1 – PF4), with PF2 being the most abundant in *P. fluorescens* BD5 cultures (Janek et al., 2010; Biniarz et al., 2018). PFs are probably synthesized by a single NRPS complex, with one of its modules lacking substrate specificity for leucine and valine (data not shown). High rate of PF2 production, together with a carefully optimized protocol for the semi-preparative HPLC purification, allowed to investigate some physicochemical and biological properties of PF2 alone (Janek et al., 2010). For example, PF2 was shown to exhibit antimicrobial properties by inhibiting adhesion and biofilm formation (Janek et al., 2012, 2016; Biniarz et al., 2015), and showed cytotoxic effects on cancer cells (Janek et al., 2013). The original protocol for the production of PFs was aimed at experimental scale production, with an efficiency of only ∼10 mg of pure PF2 per 1 L of culture in mineral salt medium (Janek et al., 2010). In our previous work we identified critical parameters essential for PFs production, mainly high glycerol and tryptone concentration, high culture aeration, and the presence of amino acids: leucine (Leu), valine (Val), or isoleucine (Ile). These experiments allowed us to develop a laboratory-scale optimized culture conditions and to achieve two goals: (1) increase PFs production ∼120-fold to meet the demand for large amounts of pure PFs needed for further experiments, and (2) separate individual PF variants, for investigating structure-properties relationship of CLPs (Biniarz et al., 2018). Simultaneously, we established a protocol for the precise quantification of PFs, together with their structural analysis by LC-MS/MS (Biniarz and Łukaszewicz, 2017).

The aim of this work was to develop a bioprocess for the increased production and purification of PFs in laboratory-scale bioreactors and then to transfer this process to technical scale. To this end, we established an efficient culturing of *P. fluorescens* BD5 in bioreactors, initially in 2.5 L and next in 30 L of working volumes, using optimized media and conditions (Biniarz et al., 2018). We also demonstrated an efficient process of raw PFs purification from the foam collected from cultures, together with the purification and separation of PF structural analogs with semi-preparative RP-HPLC. We also provided the method for the selective production of given PFs structural analogs. Potentially our methods can be applied for the production and purification of any *Pseudomonas* or *Bacillus*-derived CLPs.

## Materials and Methods

### Chemicals and Culture Media

Chemicals and media components were purchased from manufacturers as follows: tryptone (Becton Dickinson, United States); proteose peptone (Difco, United States); K_2_HPO_4_, MgSO_4_, NaOH, H_3_PO_4_ (POCH, Poland); LB, MOPS, L-leucine (Leu), and L-valine (Val) (Bioshop, Canada); glycerol (VWR International, United States).

King’s B medium (KB) composition was as follow: 10 g/L glycerol, 20 g/L proteose peptone, 1.5 g/L K_2_HPO_4_, 1.5 g/L MgSO_4_ × 7H_2_O, and 100 mM MOPS (King et al., 1954; Biniarz et al., 2018). Cultures in bioreactors were performed in KB-mod medium with Leu or Val (Biniarz et al., 2018): 80 g/L glycerol, 15 g/L tryptone, 5 g/L Leu/Val, 1.5 g/L K_2_HPO_4_, 1.5 g/L MgSO_4_ × 7H_2_O, 100 mM MOPS. The pH of all media used was set at 7.0 with 6 M NaOH or 6 M HCl prior to autoclaving.

### Strain Used in the Study

*Pseudomonas fluorescens* BD5 (PCM B/00115) originating from a glycerol stock (stored at −80°C) was grown on LB agar plates at 28°C (Janek et al., 2010). After one-day incubation, single colonies were used to inoculate 10 mL of LB medium in test tubes (1st stage precultures) and incubated overnight at 28°C with 180 rpm shaking. Next, 1st stage precultures were used to inoculate 2nd stage precultures. These were performed in 300 mL Erlenmeyer flasks, filled with 100 mL of KB medium. The 2nd stage precultures were inoculated to an initial optical density (OD) of 0.1 and incubated 20 – 24 h at 28°C (180 rpm).

### Production of Pseudofactins in a Laboratory-Scale Bioreactor

**FIGURE 1:**
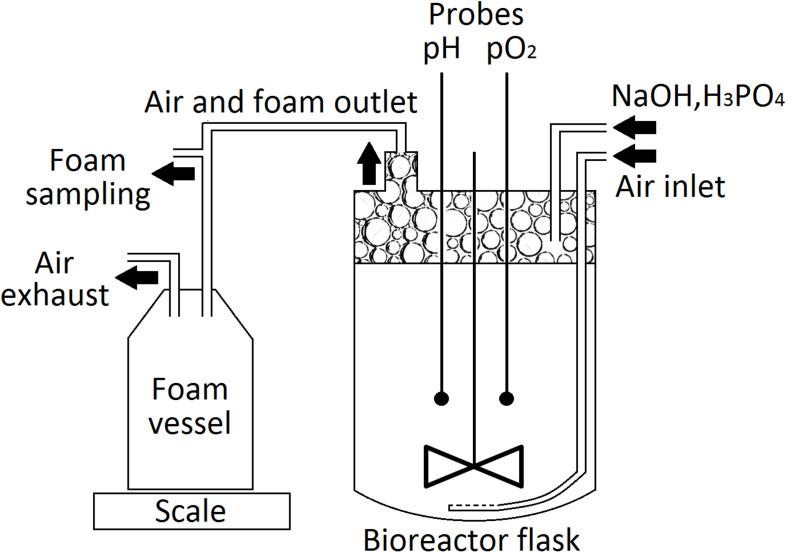
Schematic view of the bioreactor tank set-up used for the production of PFs.

Cultures for the laboratory-scale production of PFs were performed in a modified Labfors 3 (Infors HT) bioreactor (Figure 1), equipped with a 3-L glass flask. *P. fluorescens* BD5 was cultivated in 2.5 L of KB-mod medium with Leu or Val (KB-mod-Leu/Val). The cultures were inoculated with the 2nd stage precultures to the initial OD of 0.1, and then incubated at 28°C. The agitation speed was set at 300 rpm and the aeration speed at 3 L/min pH was maintained at 7.0 ± 0.1 using 3 M NaOH or 1 M H_3_PO_4_. The foam was let to overflow from the bioreactor flask through a 15 cm stainless steel tube (15 mm ID) connected to the bioreactors’ lid, followed by a silicone tube (15 mm ID) to a 5-L polypropylene, autoclaved vessel. Samples for an OD and PFs concentration measurements were aseptically collected at given time points from cultivation medium and overflowing foam. pH, pO_2_ and the weight of collected foam were measured online. All culturing experiments and processes were performed in at least three independent replicates.

### Production of Pseudofactins in a Technical-Scale Bioreactor

Cultivations in 42-L custom-designed bioreactors (ZETA Biopharma GmbH, Lieboch, Austria) were performed in 30 L of KB-mod-Leu with a minor modification. Here 25 mM MOPS instead of 100 mM MOPS was used. 10 L of the concentrated medium was transferred to the bioreactor tank, diluted to the final volume of 30 L, and then autoclaved for 20 min in 121°C. Cool medium was inoculated with the 2nd stage precultures to the OD of 0.1, and then incubated at 28°C. The agitation speed was set at 300 rpm and the aeration speed at 30 L/min The foam was let to overflow from the bioreactor tank, as described earlier. pH was maintained at 7.0 ± 0.1 using 3 M NaOH or 1 M H_3_PO_4_. Samples for OD and PFs concentration measurements were aseptically collected at given time points from both cultivation medium and overflowing foam, whereas pH and pO_2_ in culture medium were measured online. Cultivations were performed in at least two independent replicates.

### Extraction of Pseudofactins From the Collected Foam

**FIGURE 2:**
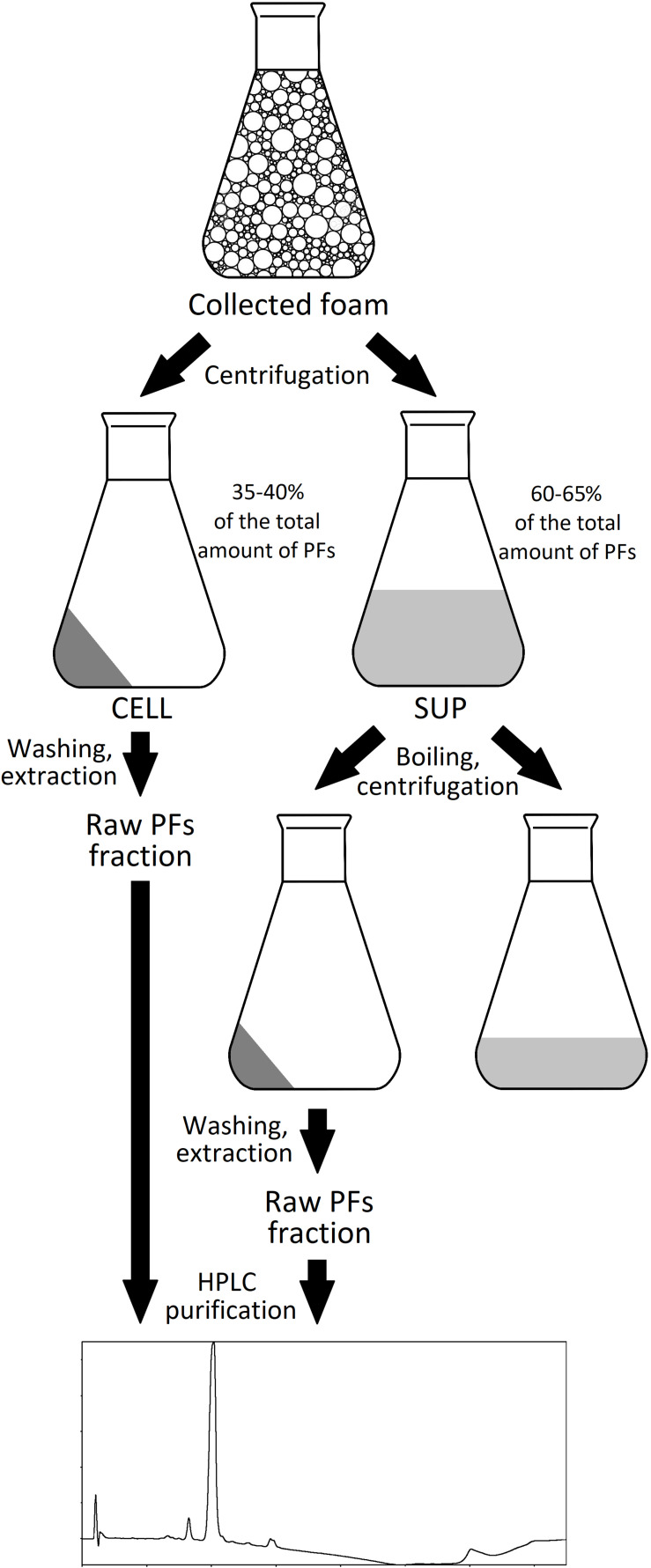
The schematic representation of PFs purification from foam.

The foam produced in the bioreactors was collected in the external tank, as shown in Figure 1. Collected foam was centrifuged (15,000 × *g*, 30 min, 4°C) and separated into two fractions: clear supernatant (SUP) and wet cell pellet (CELL). Both fractions were used for the isolation of PFs. For the CELL fraction, 40 g of it (amount of CELL in approximately 500 g of collected foam) was washed with 50 mL of deionized water and then extracted three times with 50 mL of acetonitrile (30 min, 180 rpm, 28°C). After each washing or extraction step, samples were centrifuged (15,000 × *g*, 30 min, 4°C) and extracts were collected for further analyses. For the SUP fraction, 500 mL of it was heated up in the boiling water bath for 15, 30, or 60 min and then cooled down and centrifuged (15,000 × *g*, 30 min, 4°C). Supernatants were collected and precipitate was washed with 10 mL of deionized water, followed by a triple extraction with 50 mL of methanol, ethanol, acetonitrile or ethyl acetate (30 min, 180 rpm, 28°C). Samples were centrifuged (15,000 × *g*, 30 min, 4°C) after each extraction step, clarified extracts were collected for further analyses. The schematic representation of PFs purification process is shown in Figure 2.

### Selective Production and Purification of Pseudofactin Structural Analogs

Selective production of PF structural analogs was achieved by medium supplementation with Leu or Val, as mentioned in section “Production of PFs in a laboratory-scale bioreactor” and recovered from collected foam as described in section “Extraction of PFs from the collected foam.” Obtained SUP methanolic extracts were analyzed and purified with HPLC. Analytical HPLC methods were reported earlier (Biniarz and Łukaszewicz, 2017; Biniarz et al., 2018), whereas semi-preparative HPLC purification of PF analogs is described below.

The semi-preparative HPLC system consisted of a Coulter System Gold 126 NMP Pump (Beckman) and Variable Wavelength Monitor UV/VIS detector (Knauer), running under control of the LP-Chrom software (Lipopharm, Poland). 2 mL of SUP methanol extract (approx. 10 mg/mL of dry mass) was injected onto a Phenomenex Luna C18(2) (100 × 30 mm, 10 μm) column. A 40-min gradient of 0.1% TFA in water (solvent A) and 0.1% TFA in acetonitrile (solvent B) was used (% A:B v/v): 0 min (30:70), 5 min (30:70), 10 min (10:80), 20 min (20:80), 21 min (0:100), 31 min (0:100), 32 min (30:70), 40 min (30:70). The flow rate was set to 10 mL/min and absorbance at 210 nm was monitored. Fractions were collected, freeze-dried, weighted, resuspended in methanol, and analyzed with an analytical HPLC (Biniarz and Łukaszewicz, 2017; Biniarz et al., 2018).

### Analytical Methods

Biomass concentration was evaluated by measuring the optical density (OD) at 600 nm using an Oddyssey DR/2500 (Hach, United States) or UV-3100 PC (VWR International, United States) spectrophotometers.

Pseudofactins concentration was measured using HPLC or HPTLC in cell-free culture supernatants or collapsed and clarified foam, as described previously (Geissler et al., 2016; Biniarz and Łukaszewicz, 2017). Samples were prepared prior analyses by centrifugation (15,000 × *g*, 15 min, 4°C) and then supernatants were withdrawn, diluted 10 – 100-times with methanol and centrifuged again (15,000 × *g*, 15 min, 4°C). Supernatants were used for HPLC and HPTLC analyses (Geissler et al., 2016; Biniarz and Łukaszewicz, 2017). Partial validation of the HPTLC method for PFs quantification and HPTLC analysis of PFs concentration are described in Supplementary Material.

Dry mass content was measured as follows: 10 mL of sample was freeze-dried and weighted. Dry mass was expressed as mg/mL and purity of PFs was calculated in relation to dry mass content in the samples.

PFs obtained with thermal co-precipitation were additionally analyzed with time-of-flight mass spectrometry (ToF-MS) to confirm intact PFs’ structures. An UPLC-MS system consisting of a Waters e2695 pumping module with an autosampler and a 2998 PDA detector, equipped with a Waters C18 Xbridge column (50 mm × 4.6 mm, 2.5 μm), connected to a Waters Xevo QToF MS System were used, as previously reported (Biniarz and Łukaszewicz, 2017).

### Data Analysis

A spreadsheet software (Microsoft Excel) was used to analyze the obtained data. Means, standard deviations (SD), and relative standard deviations (RSD) were calculated. All models (microbial growth, PFs production, specific growth rates and specific PFs production) were calculated with a scientific graphics and statistics software (SigmaPlot, Systat Software Inc., San Jose, United States) using sigmoidal, 3 parameter fits, as described before (Henkel et al., 2014).

## Results and Discussion

The original protocol for the production of PFs required stationary cultivation of *P. fluorescens* BD5 in mineral salt medium for 7 days, followed by clarification of cultures and supernatants extraction with ethyl acetate. PFs were then purified from raw LPs extracts with a semi-preparative RP-HPLC (Janek et al., 2010). The estimated amounts of PFs produced with this simple method were approximately 10 mg/L (Janek et al., 2010). Later, an optimized cultivation method and media for the efficient production of PFs in shaking flasks were reported. The amounts of PFs produced in intensively aerated cultures using the optimized KB-Opt medium reached 1200 mg/L, representing a 120-fold increase in comparison to the abovementioned cultures in mineral salt medium (Biniarz et al., 2018). Moreover the protocol for selective production of PF structural analogs (PF1 and PF2), by a simple supplementation of cultivation medium with Leu or Val, was provided (Biniarz et al., 2018).

To meet the requirements of high culture aeration and scaling-up the production of PFs, in this work a laboratory-scale bioprocess in a benchtop bioreactor in 2.5-L working volume and a technical-scale production in 30-L working volume were developed. The use of bioreactors allowed not only bioprocess up-scaling, but also enabled more precise monitoring and control of bioprocess parameters. Also, sampling and product removal were more straightforward than for standard microbial cultures in flasks.

### Production of Pseudofactins in Bioreactors

**FIGURE 3:**
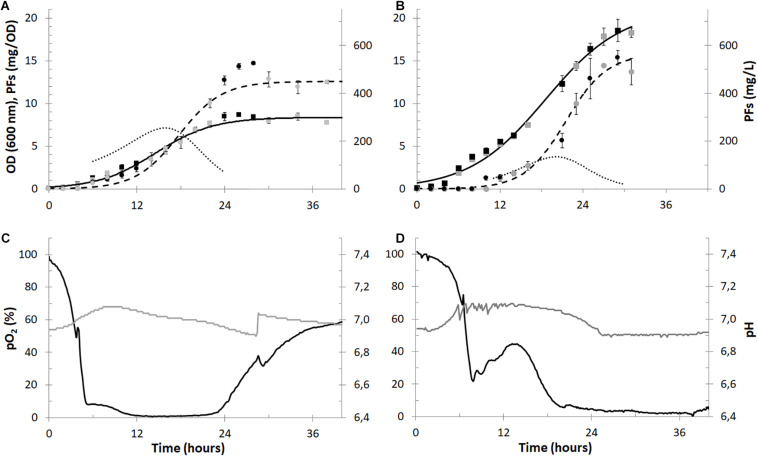
Batch cultures of *P. fluorescens* BD5 in 2.5-L (panels **A** and **C**) and 30-L (panels **B** and **D**) working volumes. Microbial growth (OD, black and gray squares), PFs production (mg/L, black and gray circles) are shown in panels **(A,B)** for the 2.5- and 30-L cultures, respectively. Alongside, respective models of microbial growth (solid black lines), PFs production (dashed black lines) and PFs specific production (mg/OD, dotted black lines) are shown in panels **(A,B)**. In panels **(C,D)** data for pO_2_ (black lines) and pH (gray lines) in cultivation medium is shown.

The laboratory-scale cultures of *P. fluorescens* BD5 were performed in 2.5 L of KB-mod medium (Figures 3A,C). During the bioprocess, pH, pO_2_ and temperature were monitored in real-time in the culture medium. In certain time-points culture medium and overflowing foam were sampled for the microbial growth (OD) and PFs concentration measurements. The semi-industrial cultures were performed in 30 L of KB-mod medium (Figures 3B,D). As we observed excessive foaming of the cultures, beginning from approximately the 6th h of the experiments, chemical and mechanical foam-disrupting agents were used (data not shown).

The maximal OD in the 30-L cultures reached approximately 18.5 at the 30th h of the cultures and was more than twice as high compared to the maximal OD in the 2.5-L cultures (∼8.5 around 26th h). The maximal PFs concentration in both set-ups (∼550 mg/L) was comparable and was reached after the 28th h of the experiments. The maximum specific PFs production (expressed in mg of PFs per OD) reached maximally 7.1 at 16th h and 3.7 at 20th h in the 2.5- and 30-L cultures, respectively (Figures 3A,C). The differences between microbial growth and specific PFs production could be probably explained by the different aeration of the cultures and/or different bioreactors’ aspect ratios and headspace volumes. In the 2.5-L cultures pO_2_ values were decreasing quickly and oxygen depletion (pO_2_ < 10%) was observed after the 5th h (Figure 3C), limiting microbial growth (Figure 3A). Here the maximal specific growth rate (μ) was only 0.040 1/h around the 12th h. In comparison, oxygen depletion was slower in the 30-L cultures (Figure 3D) and the maximum μ was 0.057 1/h around the 14th h. pO_2_ levels in the 2.5-L cultures increased after approx. 24th h of culturing, indicating low metabolic activity of bacterial cells and/or depletion of nutrients (Figure 3C). On the contrary, pO_2_ levels remained low in the 30-L cultures till the end of the cultures (Figure 3D), suggesting higher metabolic activity of bacterial cells and slower utilization of nutrients, then in the 2.5-L cultures. Due to the excessive foaming of the 30-L cultures and big amounts of foam accumulated in the bioreactors’ headspace (despite use of the foam centrifuge), sampling of cultivation medium was difficult after the 30th h of culturing (Figure 3B).

Due to the mentioned excessive foaming of culture medium, foam overflow and collection in the external tank was tested as a method for the initial purification of PFs. Previously foam overflow and foam fractionation were tested in cultures of *Bacillus* (Davis et al., 2001; Guez et al., 2007; Willenbacher et al., 2014) and *Pseudomonas* (Heyd et al., 2011; Beuker et al., 2016; Anic et al., 2018). These works revealed a foam to be highly enriched with BS.

The foam was let to overflow freely from the bioreactor vessel during the beginning of each batch from the 2.5-L cultures, as no mechanical foam disruptor was available for this set-up. At certain time-points, culture medium and overflowing foam were sampled for the microbial growth (OD) and PFs concentration measurements. Weight of the overflowing foam was monitored in real-time (Figure 1 and Supplementary Figures 1, 2). Similar set-up was also tested for the 30-L cultures. Here, foam centrifuge was used until the 15th h of the cultures and then the foam was allowed to overflow. This was dictated by the severe foaming of a non-inoculated medium and during initial stages of the cultures.

**FIGURE 4:**
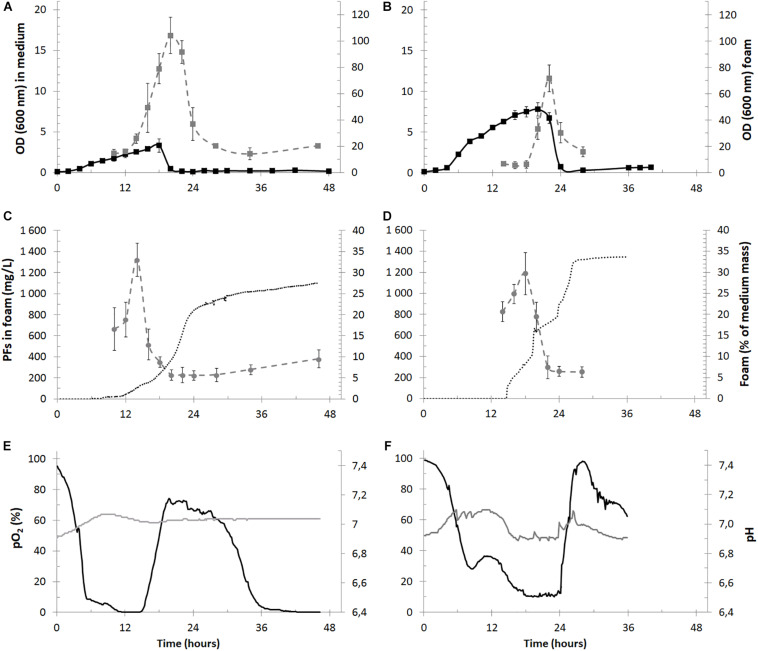
Time courses of the cultivation parameters of PFs production in bioreactors with foam overflow in the 2.5-L (panels **A,C,E**) and 30-L (panels **B,D,F**) cultures. In panels **(A,B)** measured OD in culture medium (black squares) and in overflowing foam (gray squares) are shown for the 2.5- and 30-L cultures. PFs concentration in overflowing foam (mg/L, gray circles) is shown together with a mass of collected foam (% of the initial mass of medium in bioreactor, dotted black lines) in panels **(B,D)** for the 2.5- and 30-L cultures. In panels **(E,F)** data for pO_2_ (black lines) and pH (gray lines) in cultivation medium is shown.

The laboratory-scale cultures of *P. fluorescens* BD5 were performed in 2.5 L of KB-mod medium (Figures 4A,C), whereas the semi-industrial cultures were performed in 30 L of KB-mod medium (Figures 4B,D).

The cultures reached a maximal OD of 3.32 ± 0.84 at the 18th h of the experiment and then decreased below 0.5 and remained at this level until the culture termination. Simultaneously OD measured in the overflowing foam reached its maximum of 104.1 ± 13.8 around the 20th h of culturing. Therefore, the foam in the 2.5-L cultures was enriched with bacterial cells approximately 220-fold in comparison to the culture medium at the 20th h of the experiment (Figure 4A) and the OD decrease/increase in the medium and foam, respectively, matched in time, indicating that bacterial cells were escaping the bioreactor together with foam at the height of its production (Figure 4A). Similarly, the foam was enriched with bacterial cells during the 30-L cultures. The maximal OD in the cultures reached 7.76 ± 0.84 at 20th h and 71.7 ± 10.1 in foam at 22nd h (Figure 4B).

Approximately 0.7 kg of foam was collected from the 2.5-L cultures (∼0.735 L after centrifugation) during a single bioreactor run, which was almost 30% of the initial medium mass (Figure 4C). The maximal PFs concentration in overflowing foam reached 1322.9 ± 157.4 mg/L (Figure 4C), whereas measured PFs concentration in collected foam at the end of the cultures was 511.2 ± 28.7 mg/L. The calculated quantity of PFs collected in the foam was 330 – 420 mg per bioreactor run or 132 – 170 mg of PFs per liter of the initial bioreactor volume. Simultaneously, less than 5 mg/L of PFs in the cultivation medium was detected, what shows a significant enrichment of foam with PFs. Weight of the collected foam from the 30-L cultures reached approximately 11.5 kg (∼12.1 L), what was almost 34% of the initial medium mass (Figure 4D) The maximal PFs concentration in overflowing foam reached 1186.6 ± 201.5 mg/L (Figure 4D), whereas measured PFs concentration in collected foam at the end of the cultures was 592.8 ± 43.4 mg/L. The calculated quantity of PFs collected in foam was between 6.1 and 8.3 g per bioreactor run or 202 – 279 mg of PFs per liter of the initial bioreactor volume. Only small amounts of PFs in the culture medium were detected in the 30-L cultures, with a maximum of 26.9 ± 7.4 mg/L at 8th h. This concentration decreased below 5 mg/L when foam centrifuge was turned off at 15th h and foam was allowed to overflow freely. The pO_2_ values in the 2.5-L cultures were decreasing, reaching minimal values of <10% after the 5th h of the culture. Then pO_2_ remained at low levels between 5th and 15th, suggesting high metabolic activity of *P. fluorescens* BD5. After the 15th h, pO_2_ increased to ∼70% (Figure 4E). Similar behavior was observed for the 30-L cultures (Figure 4F).

According to the literature, in the bioprocess with a continuous product removal with foam, BS production should be favored (Chen et al., 2006; Coutte et al., 2010; Santos da Silva et al., 2015; Alonso and Martin, 2016). Yet, our results suggest that non-foaming bioprocesses were more efficient in terms of the total quantity of produced PFs. For example, calculated PFs quantity in non-foaming bioprocess in 2.5-L medium reached more than 1300 mg per L (vs. 330 – 420 mg per L of PFs during foaming process). The main reason was probably the production decrease by culture dying-out, which was caused by cell removal with foam (Figures 4A,B). Moreover, it is hypothesized that efficiency of the foaming bioprocess can be also decreased due to removal of quorum sensing autoinducer molecules with foam or due to their accumulation in the hydrophobic antifoam phase (Reis et al., 2011; Henkel et al., 2013). On the other side, foam overflow can be considered as an efficient method for the initial recovery of BS from culture broth, making their further downstream processing easier and more cost-effective (Beuker et al., 2016).

### Extraction of Pseudofactins From Foam

Ultrafiltration was inefficient for the purification of PFs from foam and the reason was probably high protein concentration in the foam, as a rapid clogging of micro- and ultrafiltration membranes was observed (data not shown). Therefore, it was decided to deproteinize foam samples prior to PFs purification. It was concluded that the ideal deproteinization protocol should be industry-compatible and should not include any addition of organic solvents or salts to the foam samples. According to the literature, the addition of organic solvents or salts to BS-containing solutions may have an impact on the formation of BS micelles and, as a result, disturb further ultrafiltration process (Jauregi et al., 2013; Rangarajan et al., 2014; Janek et al., 2016). Thus, thermal denaturation of proteins was considered to be appropriate to this end.

**FIGURE 5:**
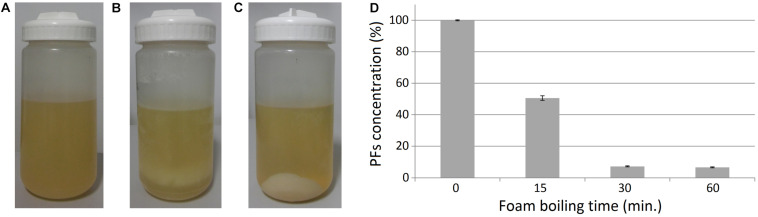
Effect of heating on the clarified foam supernatants. **(A)** Clarified foam supernatant (SUP) obtained after foam centrifugation and removal of cell pellets. **(B)** Sample boiling resulted in a formation of pellets in the samples, **(C)** which can be separated with centrifugation. **(D)** Relative amounts of PFs in supernatants after boiling. The initial concentration of PFs in the unboiled samples (0 min) was 607.7 ± 2.0 mg/L (100%).

Foam collected during the *P. fluorescens* BD5 cultures in bioreactors was centrifuged, which resulted in two fractions – wet cell pellet (CELL) and clarified foam supernatants (SUP). The SUP fractions were heated up in a boiling water bath, then cooled down and centrifuged (Figure 2). Simultaneously, PFs concentration in the clarified supernatants after boiling was monitored (Figure 5).

During the boiling step, the formation of beige pellet was observed, suggesting thermal denaturation of proteins in the samples (Figures 5A,B). The pellet could be easily separated from liquid by centrifugation (Figure 5C). Simultaneously, PFs concentration in the clarified supernatants was decreasing (Figure 5D). Even a 15-min boiling decreased PFs concentration in SUP by 49.4 ± 1.4%, from the initial value of 607.7 ± 2.0 mg/L (100%). Whereas 30- or 60-min boiling decreased PFs concentration by 92.7 ± 0.3% and 93.3 ± 0.3%, respectively (Figure 5D). These results suggest thermal degradation and/or co-precipitation of PFs with denatured proteins. Thus, it was decided to investigate the possibility of PFs thermal recovery from the SUP pellets.

In another experiment, pellets obtained after 30-min boiling of SUP fractions (containing 408.2 ± 10.7 mg/L of PFs), were centrifuged and washed with water. Then, SUP pellets were extracted three times with an organic solvents (methanol, ethanol, acetonitrile, or ethyl acetate). After each washing and extraction step, PFs and dry mass contents were quantified in resulting solutions. The experimental protocol is shown in Figure 2, while the results are shown in Table 1. Here, 6.3 ± 4.6% of the initial amount of PFs (408.2 ± 10.7 mg/L) was left in SUP after boiling. This corresponds to approximately 94% of PFs potentially precipitating during 30-min boiling. Simultaneously, significant amounts of PFs in SUP pellet extracts were detected, confirming the hypothesis of PFs co-precipitation with denatured proteins (Table 1). Methanol and acetonitrile were found to be the most effective extracting solvents. Here, even a one-step extraction allowed recovery of >85% of the initial PFs amount (>90% of PFs in pellet), and the two step extraction allowed the full recovery of PFs from pellets. Ethanol and ethyl acetate were less efficient (Table 1). Simultaneously, measured PFs purity was relatively high, reaching 63.6 ± 1.1% and 60.4 ± 2.5% for methanol and acetonitrile extracts, respectively (Table 1).

**TABLE 1:** PFs recovery (%) from foam supernatants (SUP) using proposed protocol (boiling and extraction).

		PFs recovery (%)					
		Water washing	1st Extraction	2nd Extraction	3rd Extraction	Total PFs recovery (organic fraction)	PFs purity (%)
	SUP					100.0 ± 2.6	0.7 ± 0.0
	SUP after boiling					6.3 ± 4.6	
SUP pellet extracts	Methanol	3.7 ± 0.8	88.0 ± 0.6	2.9 ± 0.2	0.0 ± 0.0	90.9 ± 0.2	63.6 ± 1.1
	Ethanol	5.9 ± 0.6	76.6 ± 2.5	8.6 ± 0.2	2.0 ± 0.2	87.2 ± 1.0	57.2 ± 2.6
	Acetonitrile	6.4 ± 0.3	85.6 ± 4.2	2.7 ± 0.1	0.0 ± 0.0	88.3 ± 2.0	60.4 ± 2.5
	Ethyl acetate	6.1 ± 0.1	56.2 ± 1.4	21.9 ± 0.5	10.7 ± 2.7	88.8 ± 0.9	45.0 ± 0.6

*Results are shown as a relative amounts of recovered PFs in comparison to PFs present in SUP (100%, 408.2 ± 10.7 mg/L).*

The scientific literature suggests an extracellular export (to the culture medium) as the main form of BS production (Najmi et al., 2018). However, some results also suggest the presence of cell-bound BS fraction (Gudiña et al., 2015). Different buffers and/or organic solvents are used for the isolation of cell-bound BS (Rodríguez et al., 2010; Gudiña et al., 2011, 2015; Vecino et al., 2015). As the large amounts of wet cell fraction (CELL) were obtained after foam centrifugation (approx. 40 *g* of CELL from 500 mL of collapsed foam), it was decided to test the possibility of extracting cell-bound PFs. Therefore, the CELL fraction was washed with water and then extracted three times with acetonitrile (Figure 2). PFs concentration and dry mass contents were tested after each washing and extraction step (Table 2).

**TABLE 2:** Recovery of the cell-bound PFs from 40 g of wet cell fraction (CELL), using proposed protocol (washing and acetonitrile extraction).

	Water washing	1st Extraction	2nd Extraction	3rd Extraction	Total PFs amount in CELL fraction
PFs (mg/L)	51.3 ± 4.7	3020.3 ± 80.6	316.6 ± 20.0	24.8 ± 4.8	
PFs (mg)	2.6 ± 0.2	151.0 ± 4.0	15.8 ± 1.0	1.2 ± 0.2	168.1 ± 4.6
PFs recovery (%)	1.5 ± 0.1	89.8 ± 2.4	9.4 ± 0.6	0.7 ± 0.1	100 ± 2.7
PFs purity (%)		14.6 ± 1.3			

*Results are shown as a relative amounts of recovered PFs in comparison to PFs present in CELL (100%, 170.6 ± 1.3 mg).*

Washing 40 g of wet cell fractions with water allowed the recovery of small amounts of PFs (2.6 ± 0.2 mg). Amount of PFs recovered in the following acetonitrile extractions were higher and reached 151.0 ± 4.0 mg and 15.8 ± 1.0 mg after 1st and 2nd extraction, respectively. The purity of obtained PFs (combined fractions 1 and 2) was low, reaching only 14.6 ± 1.3% (Table 2). Extraction of PFs from CELL fraction allowed to increase the total PFs yields from a single bioreactor run. The extracellular PFs (present in the SUP) were the major fraction, accounting for approximately 60–65% of the total amount of recovered total PFs (cf. Tables 1, 2).

The obtained results show that the proposed method of PFs extraction from foam after thermal co-precipitation can be a good alternative for other methods routinely used for preparative-scale LPs purification (e.g., acid precipitation, ultrafiltration, or solvent-solvent extraction). Obtained raw PFs fractions had an acceptable purity (>60%), and may be directly used in different applications, where lower purity or concentration is not an issue, such as plant protection or other environmental applications (Rangarajan and Clarke, 2016). The purity of raw PFs from SUP is comparable with the purity of raw LPs obtained with other purification protocols, e.g., acid precipitation or solvent-solvent extraction (Coutte et al., 2017). According to our knowledge, no similar protocol for the purification of LPs has been published so far. Simultaneously, it seems that the proposed method can be applied for the production of other LPs, potentially making their downstream processing more cost-effective and environmentally friendly. Additionally, a previously unknown fraction of cell-bounded PFs was detected, recovered and purified. Extraction of cell-bounded PFs almost doubled the overall amount of PFs purified from the bioreactor cultures.

To prove the intact structures of PFs obtained with proposed protocol, we have analyzed purified PF2 with QToF-MS system, as previously reported (Biniarz and Łukaszewicz, 2017). We have also compared the results with PF2 obtained with the original protocol by Janek et al. (2010) – culturing in mineral salt medium in shaken flasks, followed by solvent-solvent extraction and semi-preparative RP-HPLC. Our results, confirming the intact structure of PFs, can be found in Supplementary Material (Supplementary Figure 5).

### Selective Production and Purification of Pseudofactin Structural Analogs

**FIGURE 6:**
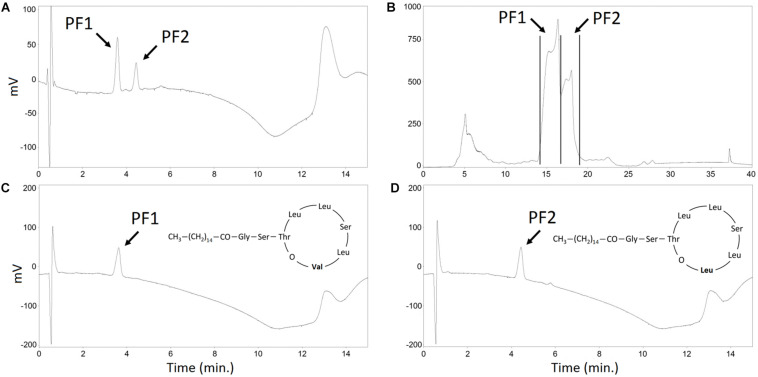
HPLC analysis of the PF structural analogs purification. **(A)** methanolic extract of SUP pellet – analytical HPLC chromatogram. **(B)** methanolic extract of SUP pellet – semi-preparative HPLC chromatogram. Collected PF1 and PF2 fractions are indicated with black lines. **(C)** Purified PF1, Rf = 3.8 min **(D)** Purified PF2, Rf = 4.2 min – analytical HPLC chromatograms. PF1 and PF2 peaks are indicated.

In the standard conditions, e.g., when cultivated in mineral salt medium, *P. fluorescens* BD5 produces mainly (>95%) PF2 analog (Janek et al., 2010). The same can be observed for the cultures in KB medium (Biniarz et al., 2018). Method for the selective production of PF1 and PF2 in baffled shake flasks was proposed earlier (Biniarz et al., 2018). The method includes supplementation of modified KB medium with amino acids – Leu or Val. Leu addition works as an inductor for PF2 production, whereas Val addition brings up the production of PF1 (Biniarz et al., 2018). A similar approach for the production of PF structural analogs in bioreactors was tested. *P. fluorescens* BD5 was cultivated in 2.5-L working volumes in a laboratory-scale bioreactor, using KB-mod-Leu or KB-mod-Val media (Biniarz et al., 2018). Next, foam was collected, heated up and extracted as described. Obtained methanolic extracts were purified with semi-preparative HPLC (Figure 6).

When KB-mod-Leu medium was used for the 2.5-L cultures, the relative abundance of PF structural analogs in collected foam was 3.9 ± 1.8% of PF1 and 96.1 ± 3.4% of PF2, with a total PFs concentration of 552.2 ± 9.5 mg/L. When KB-mod-Val medium was used for the 2.5-L cultures, the relative abundance of PF structural analogs in collected foam was 67.6 ± 3.2% of PF1 and 32.4 ± 3.0% of PF2, with a total PFs concentration of 555.5 ± 36.5 mg/L (Figure 6A). These results are comparable with the earlier report (Biniarz et al., 2018). SUP extracts were afterward purified with a semi-preparative HPLC. PF1 fraction was collected between 14.1 and 16.8 min of acetonitrile/water gradient, whereas PF2 was collected between 17.0 and 18.5 min (Figure 6B). These fractions were afterward freeze-dried, resuspended in methanol and analyzed with an analytical HPLC (Figures 6C,D) as presented in earlier reports (Biniarz and Łukaszewicz, 2017; Biniarz et al., 2018). The calculated purity of PF analogs was >95% in relation to dry mass (data not shown).

## Conclusion

A method for the production of a CLPs PFs was proposed. The production was tested in a laboratory and technical scale bioreactors. PFs were enriched in the foam overflowing from the bioreactors, which served as source material for further purification. To this end, an innovative and simple protocol for the purification of raw PFs directly from foam was presented. It includes the boiling of a supernatant, followed by extraction of the obtained precipitate. High recovery and purity levels of raw PFs were reported. Moreover, a method for the selective production of pseudofactin structural analogs, followed by their separation with a semi-preparative HPLC were proposed.

## Data Availability Statement

The original contributions presented in the study are included in the article and Supplementary Material. Further inquiries can be directed to the corresponding author.

## Author Contributions

PB: conceptualization, methodology, investigation, and formal analysis, writing – original draft, visualization, resources, and founding acquisition. MH: formal analysis, writing – review and editing, and supervision. RH: formal analysis, writing – review and editing, resources, and supervision. MŁ: conceptualization, writing – review and editing, resources, and supervision. All authors contributed to the article and approved the submitted version.

## Conflict of Interest

The authors declare that the research was conducted in the absence of any commercial or financial relationships that could be construed as a potential conflict of interest.
