# Supplementary material for: Development of a Bioprocess for the Production of Cyclic Lipopeptides Pseudofactins With Efficient Purification From Collected Foam
Source: Front Bioeng Biotechnol. 2020 Nov 23;8:565619. doi: 10.3389/fbioe.2020.565619 (PMC7719756; doi:10.3389/fbioe.2020.565619)
Supplement: Supplementary file 1 [file Data_Sheet_1.DOCX]

Supplementary Material

# Production of pseudofactins (PFs) in bioreactors


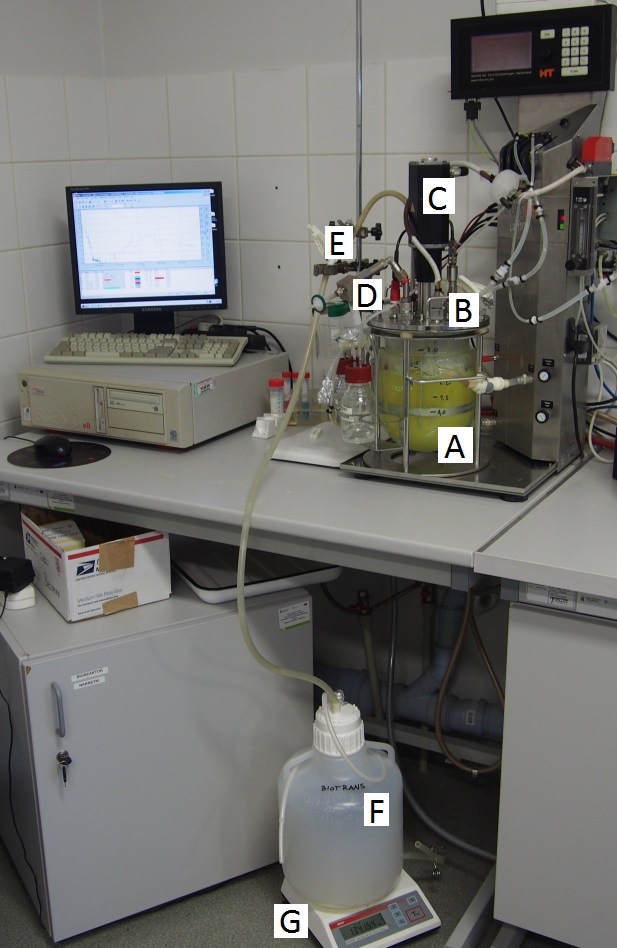


**Supplementary Figure 1.** PFs production in 2.5-L working volume. (A) bioreactor vessel, (B) pH and pO2 electrodes, (C) agitator motor, (D) bioreactor sampling port, (E) foam sampling port, (F) collected foam vessel, (G) scale.


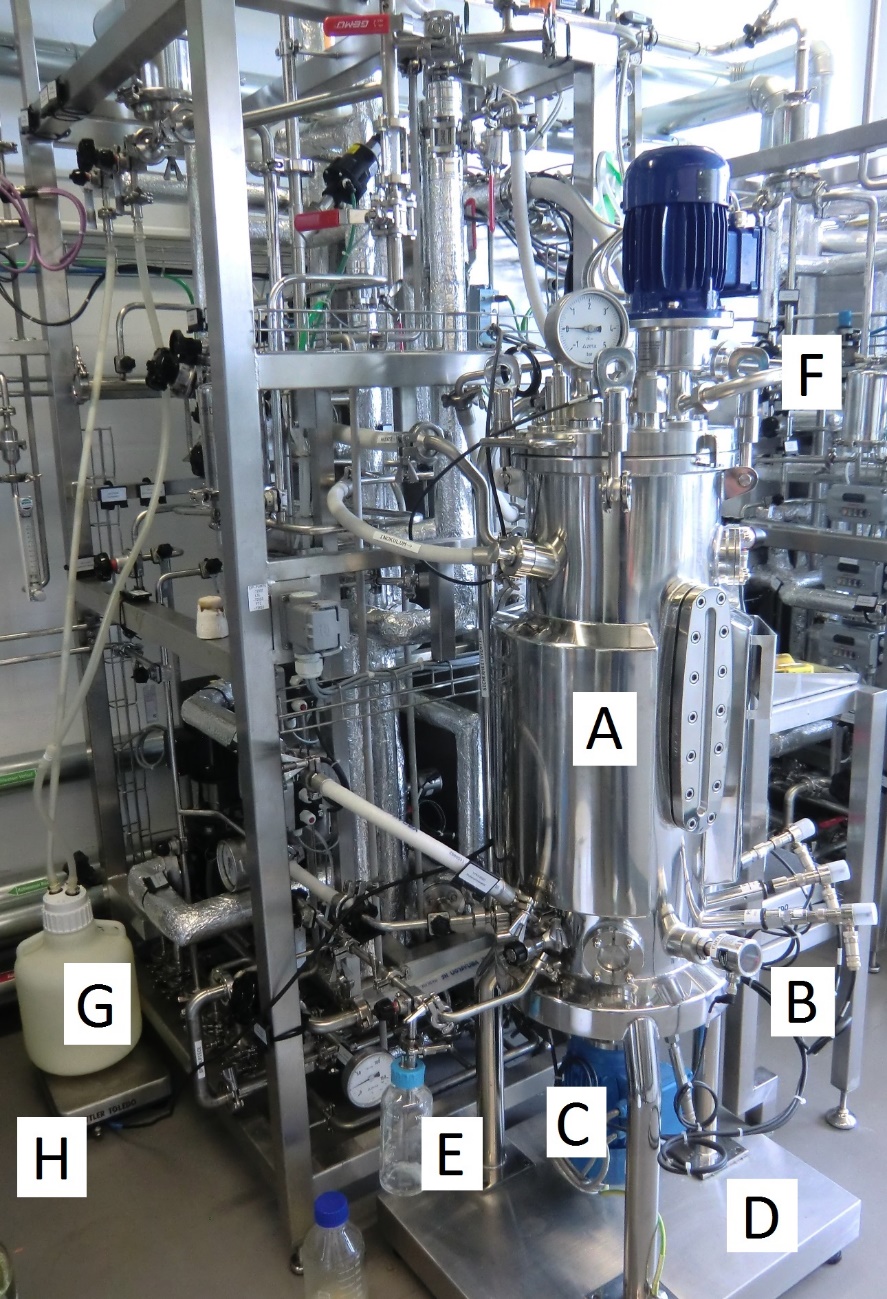


**Supplementary Figure 2.** PFs production in 30-L working volume. (A) bioreactor vessel, (B) pH and pO2 electrodes, (C) agitator motor, (D) bioreactor scale, (E) bioreactor sampling port, (F) foam sampling port, (G) collected foam vessel, (H) collected foam scale.

**HPTLC quantification of PFs**

Routinely, PFs were quantified in tested samples using HPLC (Biniarz and Łukaszewicz, 2017; Biniarz et al., 2018). For comparison, we also investigated the possibility of PFs quantification using HPTLC. HPTLC system (CAMAG, Switzerland) was used for the quantification of PFs, as it was previously shown for *Bacillus* lipopeptides (Geissler et al., 2016). The HPTLC system consisted of the Automatic TLC Sampler (ATS 4), the Automatic Developing Chamber (ADC 2), and the TLC Scanner 4. The devices were controlled by winCATS Software 1.4.7 (CAMAG). 10 µl of samples or standards were applied as 6 mm bands on Merck (Germany) HPTLC silica gel 60 plates. Plates were prewashed with methanol and dried prior use. After applying the samples, plates were developed with a mobile phase of chloroform/methanol/water (65:25:4, v/v/v), up to a migration distance of 70 mm. Then, plates were dried for 2 min and scanned at 190 nm. Areas of detected PFs peaks were compared to the PFs standard curve (Biniarz and Łukaszewicz, 2017; Geissler et al., 2016). Exemplary HPTLC chromatograms of PFs standard solution and *P. fluorescens* BD5 culture supernatant are presented in Fig. S3. HPTLC was used for the PFs quantification during production in 42-L bioreactors.


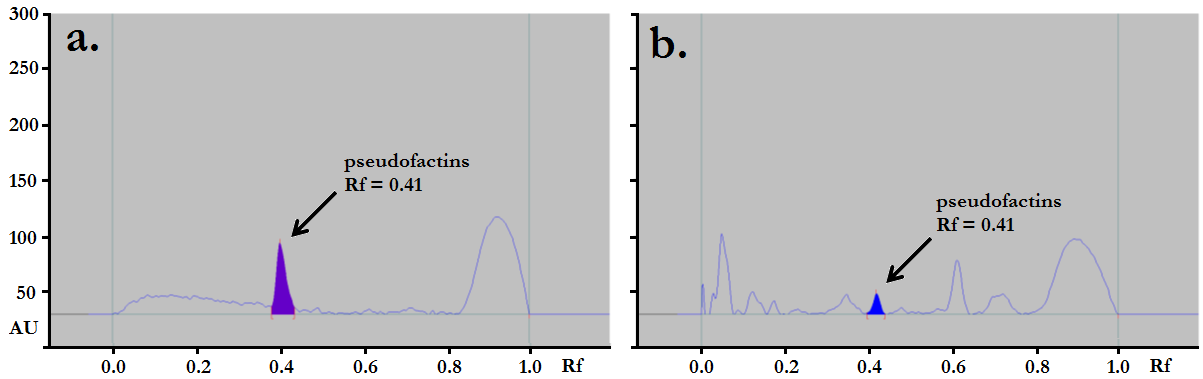


**Supplementary Figure 3.** HPTLC chromatograms of (A) PFs standard solution (0.1 mg/mL in methanol) and (B) *P. fluorescens* BD5 culture supernatant (diluted 10-times with methanol). PFs peaks are marked.

In contrast to HPLC methods, where PF structural analogues can be easily separated and detected (Biniarz and Łukaszewicz, 2017; Biniarz et al., 2018), PFs are visible as a single peak on the HPTLC chromatograms (Fig. S3). This allows estimation of total PFs concentration only. The Rf of PFs in a mobile phase of chloroform/methanol/water (65:25:4, v/v/v) was approx. 0.41 (Fig. S3). The method allows quantitation up to 11 individual samples (each in two replicates) on a standard 20 × 10 cm HPTLC plates, with a total runtime of approximately 45 minutes per plate. With a proposed HPTLC method we were able to quantify PFs in a concentration range of 30 – 300 ng PFs per spot (Fig. S4). The relative standard deviation (RSD) of these measurements was lower than 4.5% (9 replicates of each measurement).


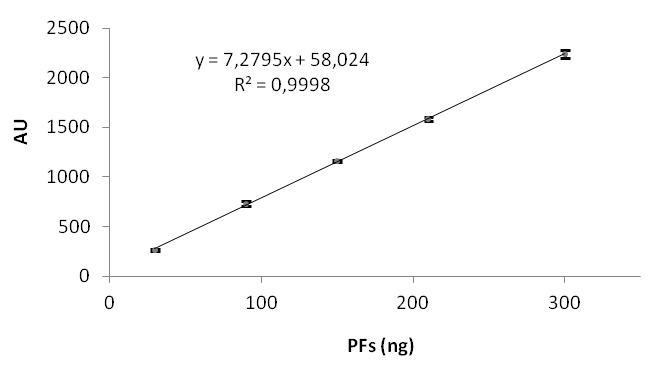


**Supplementary Figure 4.** Calibration curve for PFs quantification with HPTLC.

For the comparison, PFs in three different *P. fluorescens* BD5 culture supernatants were quantified with HPTLC and HPLC. Both methods showed acceptable levels of RSD and obtained results (PFs concentration) were comparable (Tab. S1).

**Supplementary Table 1.** PFs quantified in *P. fluorescens* BD5 culture supernatants using HPTLC and HPLC.

|  | HPTLC | | HPLC | |
| --- | --- | --- | --- | --- |
|  | PFs [mg/L] | RSD [%] | PFs [mg/L] | RSD [%] |
| Sample 1 | 434.7 ± 35.5 | 8.2 | 459.4 ± 23.8 | 5.2 |
| Sample 2 | 734.0 ± 24.6 | 3.4 | 727.0 ± 23.5 | 3.2 |
| Sample 3 | 1045.2 ± 11.7 | 1.1 | 1066.4 ± 38.9 | 3.7 |

**Time-of-Flight Mass Spectrometry analysis of purified PFs**

To prove the intact structures of PFs obtained with proposed protocol, we have analyzed purified PF2 with QToF-MS system, as previously reported (Biniarz and Łukaszewicz, 2017). We have also compared the results with PF2 obtained with the original protocol by (Janek et al., 2010) – culturing in mineral salt medium in shaken flasks, followed by solvent-solvent extraction and semi-preparative RP-HPLC. Our results confirm the intact structure of PFs (Supplementary Figure 5).


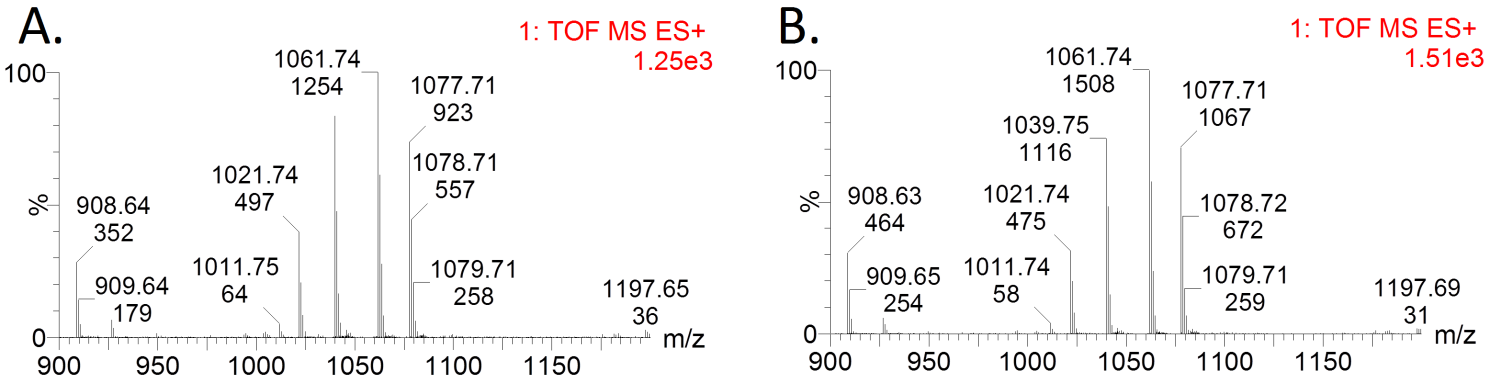


**Supplementary Figure 5.** ToF-MS spectra of PF2 obtained with the original protocol (A.) by (Janek et al., 2010) with PF2 obtained with the thermal co-precipitation. Following PF2 pseudomolecular ions can be observed: 1021.7 m/z [M+H−H2O]^+^; 1039.7 m/z [M+H]^+^; 1061.7 m/z [M+Na]^+^; 1077.7 m/z [M+K]^+^.

**Supplementary references**

Biniarz, P. et al. (2018) ‘High-throughput optimization of medium components and culture conditions for the efficient production of a lipopeptide pseudofactin by Pseudomonas fluorescens BD5’, *Microbial cell factories*, 17(121).

Biniarz, P. and Łukaszewicz, M. (2017) ‘Direct quantification of lipopeptide biosurfactants in biological samples via HPLC and UPLC-MS requires sample modification with an organic solvent’, *Applied Microbiology and Biotechnology*, 101(11), pp. 4747–4759. doi: 10.1007/s00253-017-8272-y.

Geissler, M. et al. (2016) ‘High-performance thin-layer chromatography (HPTLC) for the simultaneous quantification of the cyclic lipopeptides Surfactin, Iturin A and Fengycin in culture samples of Bacillus species’, *Journal of Chromatography B*. Elsevier B.V. doi: 10.1016/j.jchromb.2016.11.013.

Janek, T., Łukaszewicz, M., Rezanka, T., and Krasowska, A. (2010). Isolation and characterization of two new lipopeptide biosurfactants produced by *Pseudomonas fluorescens* BD5 isolated from water from the Arctic Archipelago of Svalbard. *Bioresource Technology* 101, 6118–23. doi:10.1016/j.biortech.2010.02.109.
